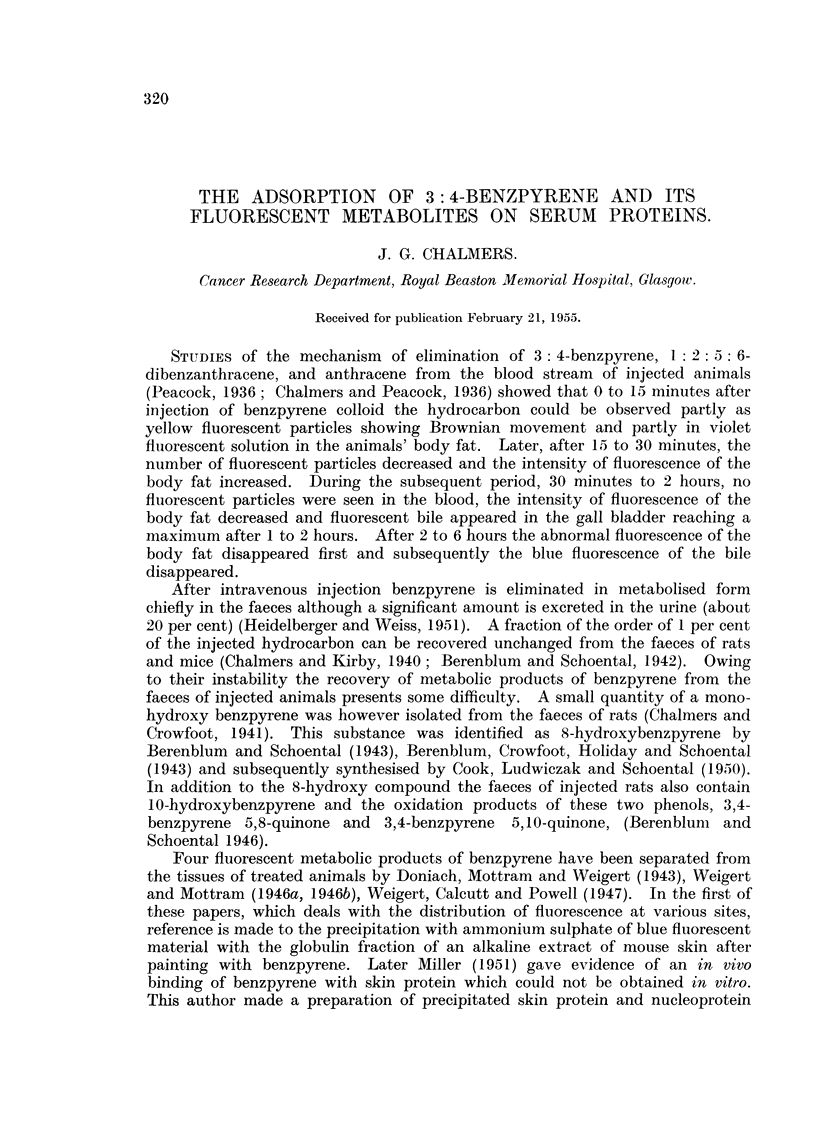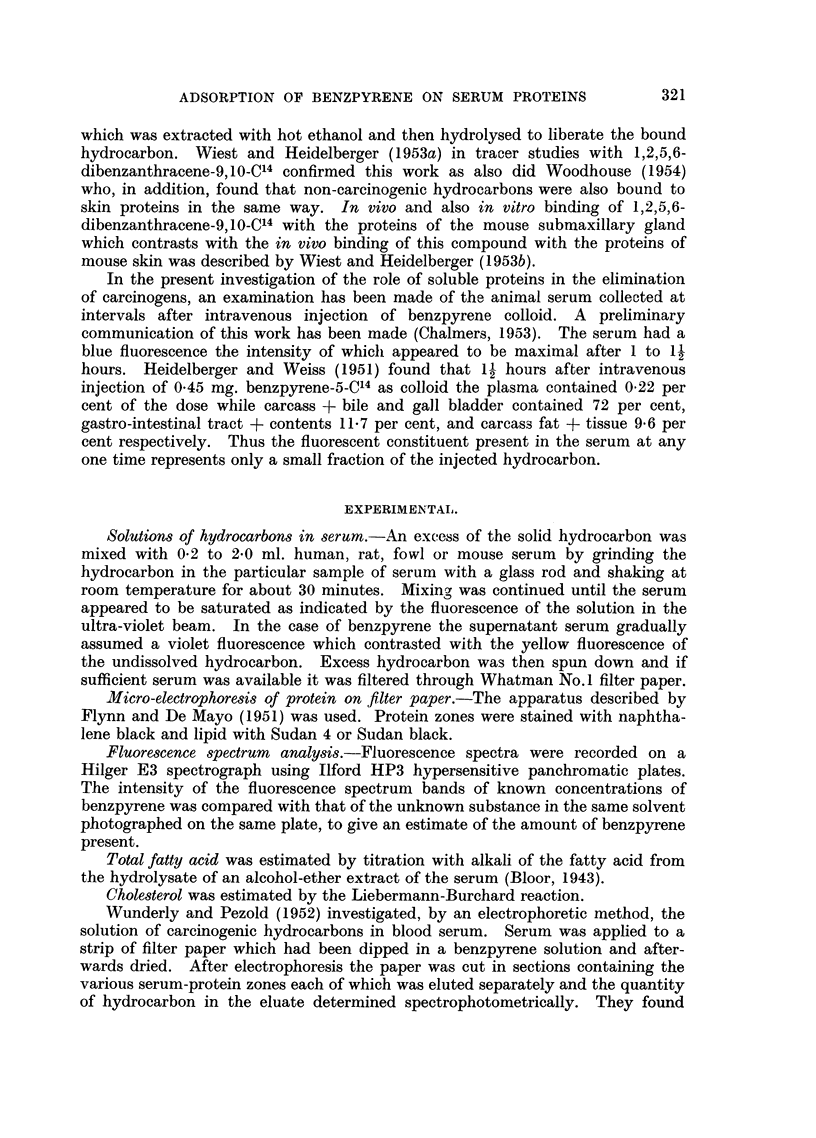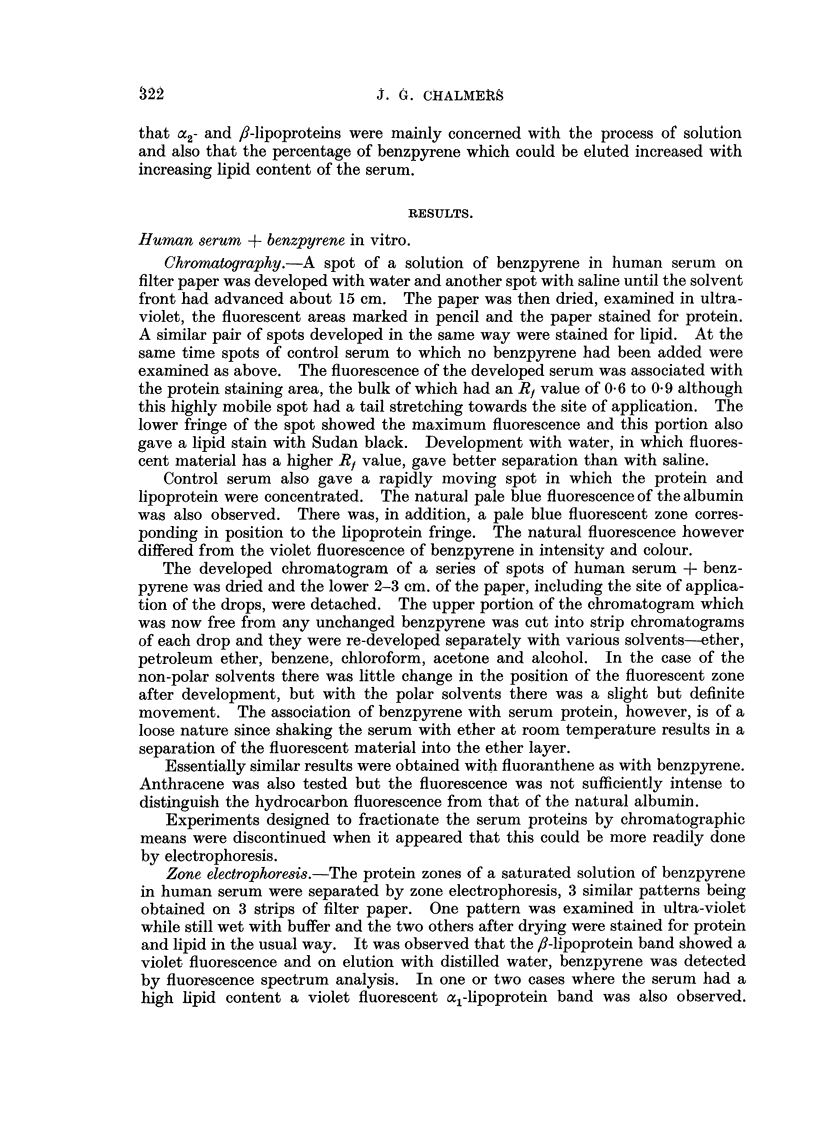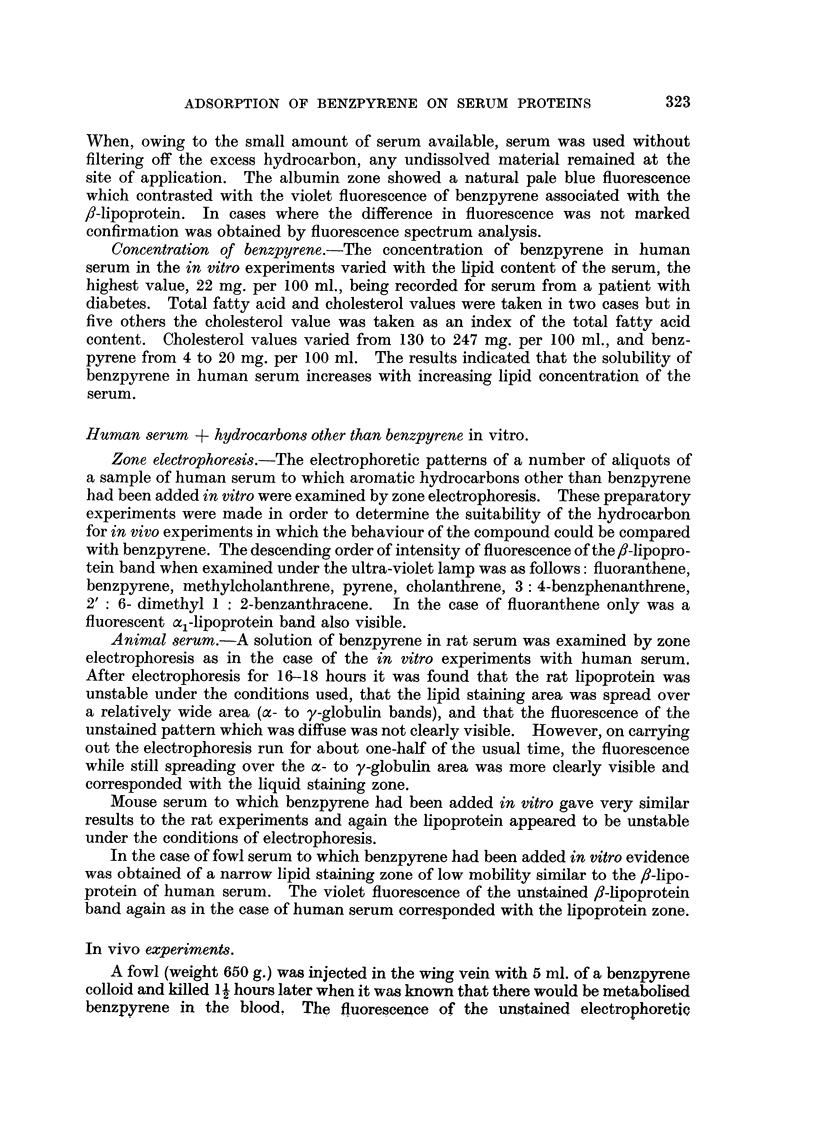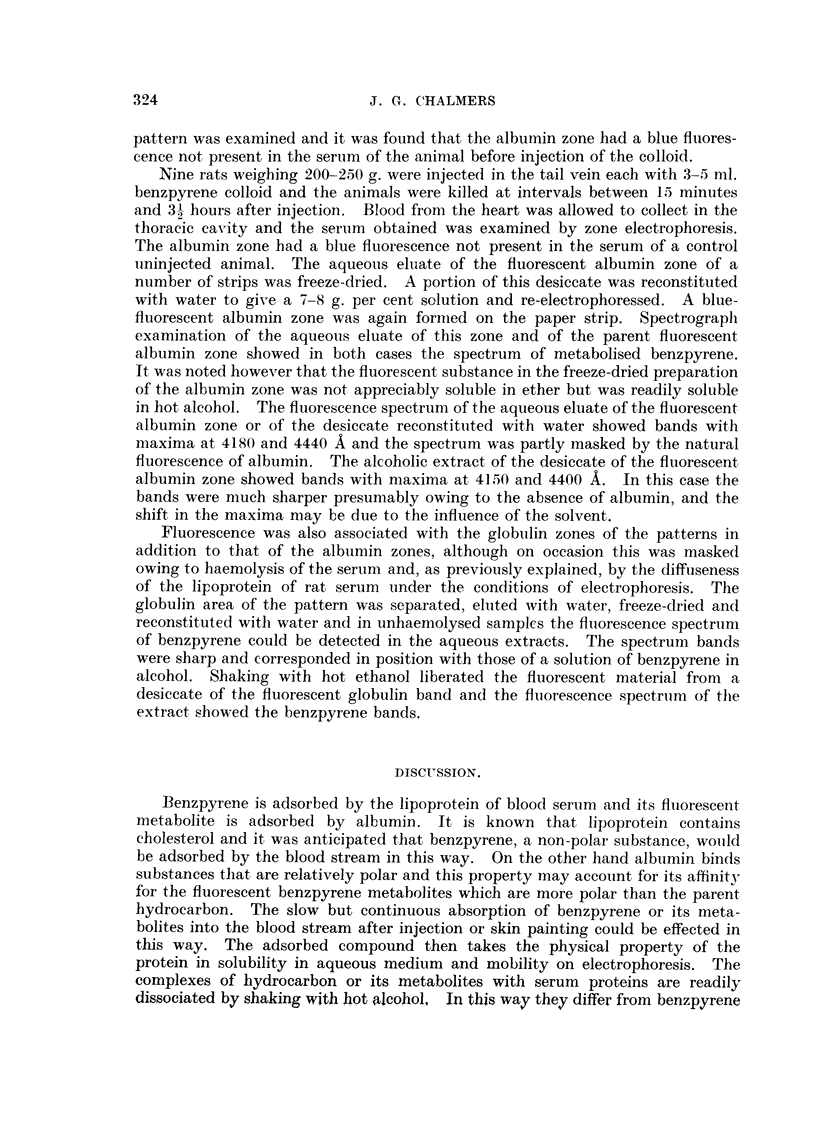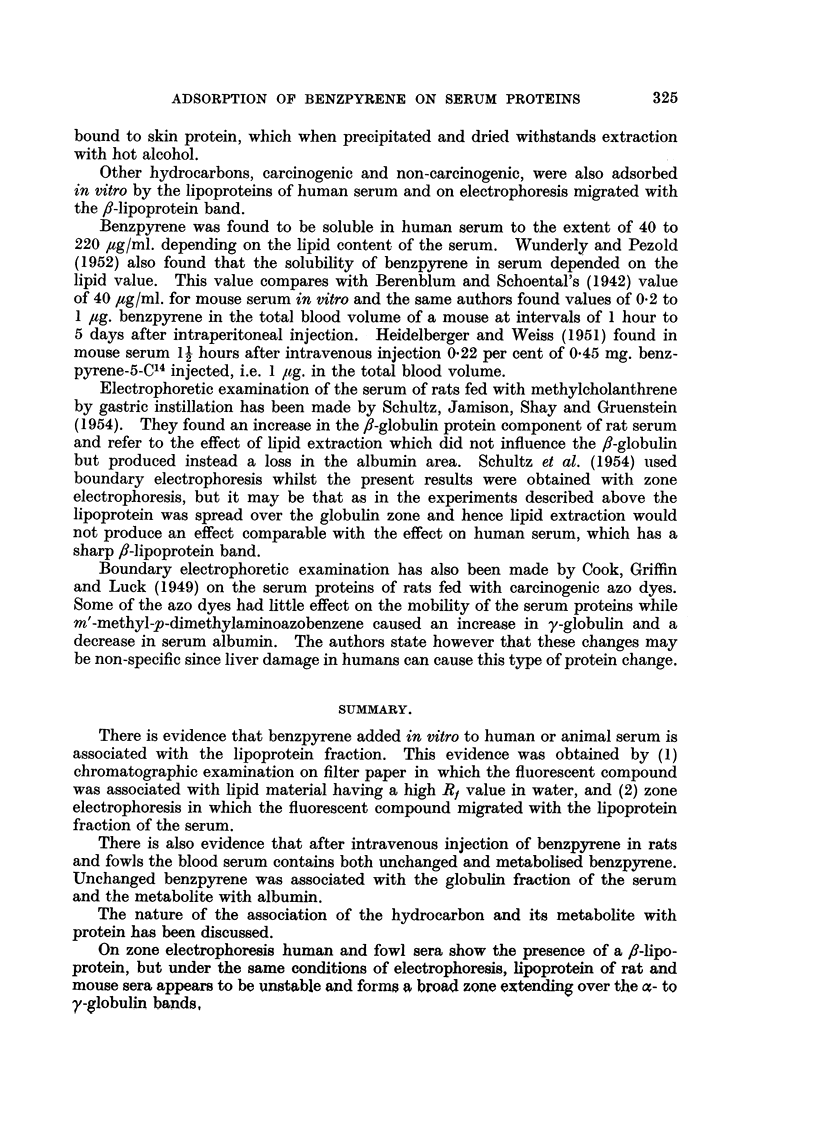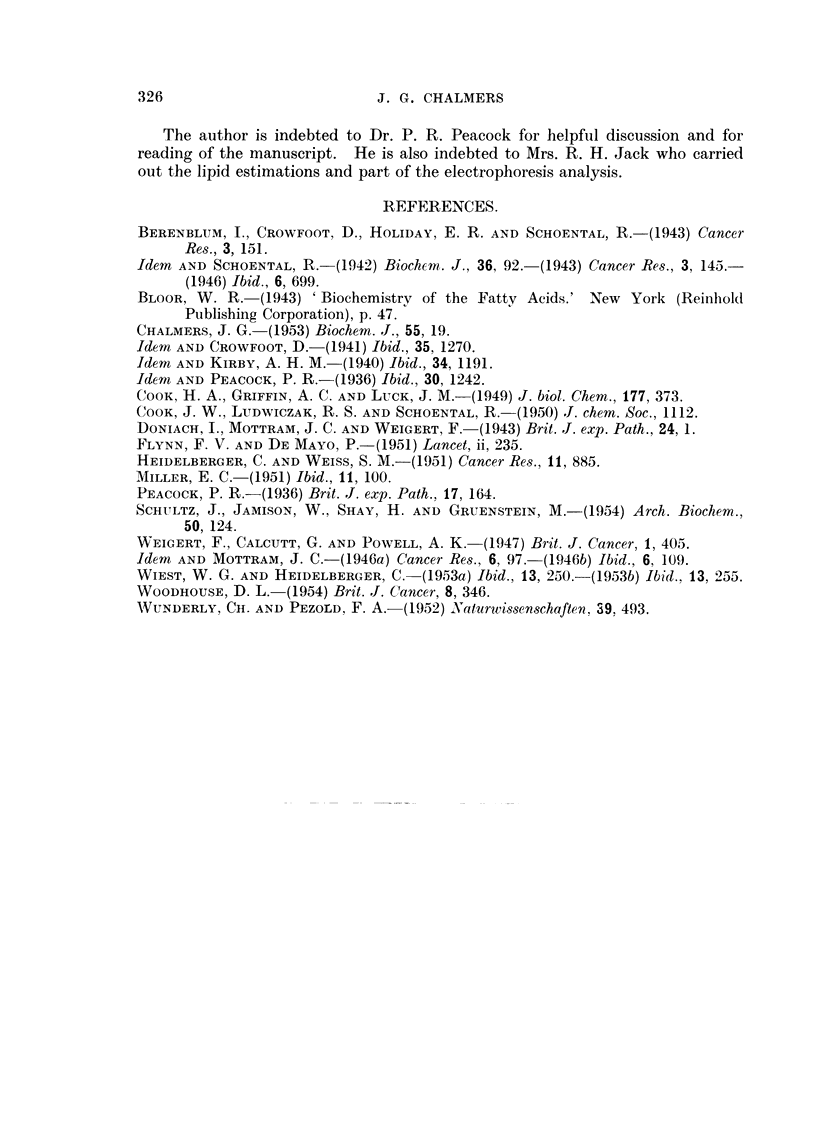# The Adsorption of 3:4-Benzpyrene and its Fluorescent Metabolites on Serum Proteins

**DOI:** 10.1038/bjc.1955.29

**Published:** 1955-06

**Authors:** J. G. Chalmers


### THE ADSORPTION OF 3 : 4-BENZPYRENE AND ITS FLUORESCENT METABOLITES ON SERUM PROTEINS.

J. G. CHALMERS.

*Cancer Research Department, Royal Beaston Memorial Hospital, Glasgow.*

Received for publication February 21, 1955.

STUDIES of the mechanism of elimination of 3 : 4-benzpyrene, 1 : 2 : 5 : 6-dibenzanthracene, and anthracene from the blood stream of injected animals (Peacock, 1936 ; Chalmers and Peacock, 1936) showed that 0 to 15 minutes after injection of benzpyrene colloid the hydrocarbon could be observed partly as yellow fluorescent particles showing Brownian movement and partly in violet fluorescent solution in the animals' body fat. Later, after 15 to 30 minutes, the number of fluorescent particles decreased and the intensity of fluorescence of the body fat increased. During the subsequent period, 30 minutes to 2 hours, no fluorescent particles were seen in the blood, the intensity of fluorescence of the body fat decreased and fluorescent bile appeared in the gall bladder reaching a maximum after 1 to 2 hours. After 2 to 6 hours the abnormal fluorescence of the body fat disappeared first and subsequently the blue fluorescence of the bile disappeared.

After intravenous injection benzpyrene is eliminated in metabolised form chiefly in the faeces although a significant amount is excreted in the urine (about 20 per cent) (Heidelberger and Weiss, 1951). A fraction of the order of 1 per cent of the injected hydrocarbon can be recovered unchanged from the faeces of rats and mice (Chalmers and Kirby, 1940 ; Berenblum and Schoental, 1942). Owing to their instability the recovery of metabolic products of benzpyrene from the faeces of injected animals presents some difficulty. A small quantity of a mono-hydroxy benzpyrene was however isolated from the faeces of rats (Chalmers and Crowfoot, 1941). This substance was identified as 8-hydroxybenzpyrene by Berenblum and Schoental (1943), Berenblum, Crowfoot, Holiday and Schoental (1943) and subsequently synthesised by Cook, Ludwiczak and Schoental (1950). In addition to the 8-hydroxy compound the faeces of injected rats also contain 10-hydroxybenzpyrene and the oxidation products of these two phenols, 3,4-benzpyrene 5,8-quinone and 3,4-benzpyrene 5,10-quinone, (Berenblum and Schoental 1946).

Four fluorescent metabolic products of benzpyrene have been separated from the tissues of treated animals by Doniach, Mottram and Weigert (1943), Weigert and Mottram (1946*a*, 1946*b*), Weigert, Calcutt and Powell (1947). In the first of these papers, which deals with the distribution of fluorescence at various sites, reference is made to the precipitation with ammonium sulphate of blue fluorescent material with the globulin fraction of an alkaline extract of mouse skin after painting with benzpyrene. Later Miller (1951) gave evidence of an *in vivo* binding of benzpyrene with skin protein which could not be obtained *in vitro*. This author made a preparation of precipitated skin protein and nucleoprotein which was extracted with hot ethanol and then hydrolysed to liberate the bound hydrocarbon. Wiest and Heidelberger (1953*a*) in tracer studies with 1,2,5,6-dibenzanthracene-9,10-$C^{14}$ confirmed this work as also did Woodhouse (1954) who, in addition, found that non-carcinogenic hydrocarbons were also bound to skin proteins in the same way. *In vivo* and also *in vitro* binding of 1,2,5,6-dibenzanthracene-9,10-$C^{14}$ with the proteins of the mouse submaxillary gland which contrasts with the *in vivo* binding of this compound with the proteins of mouse skin was described by Wiest and Heidelberger (1953*b*).

In the present investigation of the role of soluble proteins in the elimination of carcinogens, an examination has been made of the animal serum collected at intervals after intravenous injection of benzpyrene colloid. A preliminary communication of this work has been made (Chalmers, 1953). The serum had a blue fluorescence the intensity of which appeared to be maximal after 1 to 1½ hours. Heidelberger and Weiss (1951) found that 1½ hours after intravenous injection of 0·45 mg. benzpyrene-5-$C^{14}$ as colloid the plasma contained 0·22 per cent of the dose while carcass + bile and gall bladder contained 72 per cent, gastro-intestinal tract + contents 11·7 per cent, and carcass fat + tissue 9·6 per cent respectively. Thus the fluorescent constituent present in the serum at any one time represents only a small fraction of the injected hydrocarbon.

### EXPERIMENTAL.

*Solutions of hydrocarbons in serum.*—An excess of the solid hydrocarbon was mixed with 0·2 to 2·0 ml. human, rat, fowl or mouse serum by grinding the hydrocarbon in the particular sample of serum with a glass rod and shaking at room temperature for about 30 minutes. Mixing was continued until the serum appeared to be saturated as indicated by the fluorescence of the solution in the ultra-violet beam. In the case of benzpyrene the supernatant serum gradually assumed a violet fluorescence which contrasted with the yellow fluorescence of the undissolved hydrocarbon. Excess hydrocarbon was then spun down and if sufficient serum was available it was filtered through Whatman No.1 filter paper.

*Micro-electrophoresis of protein on filter paper.*—The apparatus described by Flynn and De Mayo (1951) was used. Protein zones were stained with naphthalene black and lipid with Sudan 4 or Sudan black.

*Fluorescence spectrum analysis.*—Fluorescence spectra were recorded on a Hilger E3 spectrograph using Ilford HP3 hypersensitive panchromatic plates. The intensity of the fluorescence spectrum bands of known concentrations of benzpyrene was compared with that of the unknown substance in the same solvent photographed on the same plate, to give an estimate of the amount of benzpyrene present.

*Total fatty acid* was estimated by titration with alkali of the fatty acid from the hydrolysate of an alcohol-ether extract of the serum (Bloor, 1943).

*Cholesterol* was estimated by the Liebermann-Burchard reaction.

Wunderly and Pezold (1952) investigated, by an electrophoretic method, the solution of carcinogenic hydrocarbons in blood serum. Serum was applied to a strip of filter paper which had been dipped in a benzpyrene solution and afterwards dried. After electrophoresis the paper was cut in sections containing the various serum-protein zones each of which was eluted separately and the quantity of hydrocarbon in the eluate determined spectrophotometrically. They found that $\alpha_2$- and $\beta$-lipoproteins were mainly concerned with the process of solution and also that the percentage of benzpyrene which could be eluted increased with increasing lipid content of the serum.

### RESULTS.

*Human serum + benzpyrene* in vitro.

*Chromatography.*—A spot of a solution of benzpyrene in human serum on filter paper was developed with water and another spot with saline until the solvent front had advanced about 15 cm. The paper was then dried, examined in ultra-violet, the fluorescent areas marked in pencil and the paper stained for protein. A similar pair of spots developed in the same way were stained for lipid. At the same time spots of control serum to which no benzpyrene had been added were examined as above. The fluorescence of the developed serum was associated with the protein staining area, the bulk of which had an $R_f$ value of 0·6 to 0·9 although this highly mobile spot had a tail stretching towards the site of application. The lower fringe of the spot showed the maximum fluorescence and this portion also gave a lipid stain with Sudan black. Development with water, in which fluorescent material has a higher $R_f$ value, gave better separation than with saline.

Control serum also gave a rapidly moving spot in which the protein and lipoprotein were concentrated. The natural pale blue fluorescence of the albumin was also observed. There was, in addition, a pale blue fluorescent zone corresponding in position to the lipoprotein fringe. The natural fluorescence however differed from the violet fluorescence of benzpyrene in intensity and colour.

The developed chromatogram of a series of spots of human serum + benzpyrene was dried and the lower 2–3 cm. of the paper, including the site of application of the drops, were detached. The upper portion of the chromatogram which was now free from any unchanged benzpyrene was cut into strip chromatograms of each drop and they were re-developed separately with various solvents—ether, petroleum ether, benzene, chloroform, acetone and alcohol. In the case of the non-polar solvents there was little change in the position of the fluorescent zone after development, but with the polar solvents there was a slight but definite movement. The association of benzpyrene with serum protein, however, is of a loose nature since shaking the serum with ether at room temperature results in a separation of the fluorescent material into the ether layer.

Essentially similar results were obtained with fluoranthene as with benzpyrene. Anthracene was also tested but the fluorescence was not sufficiently intense to distinguish the hydrocarbon fluorescence from that of the natural albumin.

Experiments designed to fractionate the serum proteins by chromatographic means were discontinued when it appeared that this could be more readily done by electrophoresis.

*Zone electrophoresis.*—The protein zones of a saturated solution of benzpyrene in human serum were separated by zone electrophoresis, 3 similar patterns being obtained on 3 strips of filter paper. One pattern was examined in ultra-violet while still wet with buffer and the two others after drying were stained for protein and lipid in the usual way. It was observed that the $\beta$-lipoprotein band showed a violet fluorescence and on elution with distilled water, benzpyrene was detected by fluorescence spectrum analysis. In one or two cases where the serum had a high lipid content a violet fluorescent $\alpha_1$-lipoprotein band was also observed.

When, owing to the small amount of serum available, serum was used without filtering off the excess hydrocarbon, any undissolved material remained at the site of application. The albumin zone showed a natural pale blue fluorescence which contrasted with the violet fluorescence of benzpyrene associated with the $\beta$-lipoprotein. In cases where the difference in fluorescence was not marked confirmation was obtained by fluorescence spectrum analysis.

*Concentration of benzpyrene.*—The concentration of benzpyrene in human serum in the *in vitro* experiments varied with the lipid content of the serum, the highest value, 22 mg. per 100 ml., being recorded for serum from a patient with diabetes. Total fatty acid and cholesterol values were taken in two cases but in five others the cholesterol value was taken as an index of the total fatty acid content. Cholesterol values varied from 130 to 247 mg. per 100 ml., and benzpyrene from 4 to 20 mg. per 100 ml. The results indicated that the solubility of benzpyrene in human serum increases with increasing lipid concentration of the serum.

*Human serum + hydrocarbons other than benzpyrene* in vitro.

*Zone electrophoresis.*—The electrophoretic patterns of a number of aliquots of a sample of human serum to which aromatic hydrocarbons other than benzpyrene had been added *in vitro* were examined by zone electrophoresis. These preparatory experiments were made in order to determine the suitability of the hydrocarbon for *in vivo* experiments in which the behaviour of the compound could be compared with benzpyrene. The descending order of intensity of fluorescence of the $\beta$-lipoprotein band when examined under the ultra-violet lamp was as follows: fluoranthene, benzpyrene, methylcholanthrene, pyrene, cholanthrene, 3 : 4-benzphenanthrene, 2′ : 6- dimethyl 1 : 2-benzanthracene. In the case of fluoranthene only was a fluorescent $\alpha_1$-lipoprotein band also visible.

*Animal serum.*—A solution of benzpyrene in rat serum was examined by zone electrophoresis as in the case of the *in vitro* experiments with human serum. After electrophoresis for 16–18 hours it was found that the rat lipoprotein was unstable under the conditions used, that the lipid staining area was spread over a relatively wide area ($\alpha$- to $\gamma$-globulin bands), and that the fluorescence of the unstained pattern which was diffuse was not clearly visible. However, on carrying out the electrophoresis run for about one-half of the usual time, the fluorescence while still spreading over the $\alpha$- to $\gamma$-globulin area was more clearly visible and corresponded with the liquid staining zone.

Mouse serum to which benzpyrene had been added *in vitro* gave very similar results to the rat experiments and again the lipoprotein appeared to be unstable under the conditions of electrophoresis.

In the case of fowl serum to which benzpyrene had been added *in vitro* evidence was obtained of a narrow lipid staining zone of low mobility similar to the $\beta$-lipoprotein of human serum. The violet fluorescence of the unstained $\beta$-lipoprotein band again as in the case of human serum corresponded with the lipoprotein zone.

In vivo *experiments.*

A fowl (weight 650 g.) was injected in the wing vein with 5 ml. of a benzpyrene colloid and killed 1½ hours later when it was known that there would be metabolised benzpyrene in the blood. The fluorescence of the unstained electrophoretic pattern was examined and it was found that the albumin zone had a blue fluorescence not present in the serum of the animal before injection of the colloid.

Nine rats weighing 200–250 g. were injected in the tail vein each with 3–5 ml. benzpyrene colloid and the animals were killed at intervals between 15 minutes and $3\frac{1}{2}$ hours after injection. Blood from the heart was allowed to collect in the thoracic cavity and the serum obtained was examined by zone electrophoresis. The albumin zone had a blue fluorescence not present in the serum of a control uninjected animal. The aqueous eluate of the fluorescent albumin zone of a number of strips was freeze-dried. A portion of this desiccate was reconstituted with water to give a 7–8 g. per cent solution and re-electrophoressed. A blue-fluorescent albumin zone was again formed on the paper strip. Spectrograph examination of the aqueous eluate of this zone and of the parent fluorescent albumin zone showed in both cases the spectrum of metabolised benzpyrene. It was noted however that the fluorescent substance in the freeze-dried preparation of the albumin zone was not appreciably soluble in ether but was readily soluble in hot alcohol. The fluorescence spectrum of the aqueous eluate of the fluorescent albumin zone or of the desiccate reconstituted with water showed bands with maxima at 4180 and 4440 Å and the spectrum was partly masked by the natural fluorescence of albumin. The alcoholic extract of the desiccate of the fluorescent albumin zone showed bands with maxima at 4150 and 4400 Å. In this case the bands were much sharper presumably owing to the absence of albumin, and the shift in the maxima may be due to the influence of the solvent.

Fluorescence was also associated with the globulin zones of the patterns in addition to that of the albumin zones, although on occasion this was masked owing to haemolysis of the serum and, as previously explained, by the diffuseness of the lipoprotein of rat serum under the conditions of electrophoresis. The globulin area of the pattern was separated, eluted with water, freeze-dried and reconstituted with water and in unhaemolysed samples the fluorescence spectrum of benzpyrene could be detected in the aqueous extracts. The spectrum bands were sharp and corresponded in position with those of a solution of benzpyrene in alcohol. Shaking with hot ethanol liberated the fluorescent material from a desiccate of the fluorescent globulin band and the fluorescence spectrum of the extract showed the benzpyrene bands.

### DISCUSSION.

Benzpyrene is adsorbed by the lipoprotein of blood serum and its fluorescent metabolite is adsorbed by albumin. It is known that lipoprotein contains cholesterol and it was anticipated that benzpyrene, a non-polar substance, would be adsorbed by the blood stream in this way. On the other hand albumin binds substances that are relatively polar and this property may account for its affinity for the fluorescent benzpyrene metabolites which are more polar than the parent hydrocarbon. The slow but continuous absorption of benzpyrene or its metabolites into the blood stream after injection or skin painting could be effected in this way. The adsorbed compound then takes the physical property of the protein in solubility in aqueous medium and mobility on electrophoresis. The complexes of hydrocarbon or its metabolites with serum proteins are readily dissociated by shaking with hot alcohol. In this way they differ from benzpyrene bound to skin protein, which when precipitated and dried withstands extraction with hot alcohol.

Other hydrocarbons, carcinogenic and non-carcinogenic, were also adsorbed *in vitro* by the lipoproteins of human serum and on electrophoresis migrated with the $\beta$-lipoprotein band.

Benzpyrene was found to be soluble in human serum to the extent of 40 to 220 $\mu$g/ml. depending on the lipid content of the serum. Wunderly and Pezold (1952) also found that the solubility of benzpyrene in serum depended on the lipid value. This value compares with Berenblum and Schoental's (1942) value of 40 $\mu$g/ml. for mouse serum *in vitro* and the same authors found values of 0·2 to 1 $\mu$g. benzpyrene in the total blood volume of a mouse at intervals of 1 hour to 5 days after intraperitoneal injection. Heidelberger and Weiss (1951) found in mouse serum $1\frac{1}{2}$ hours after intravenous injection 0·22 per cent of 0·45 mg. benzpyrene-5-$C^{14}$ injected, i.e. 1 $\mu$g. in the total blood volume.

Electrophoretic examination of the serum of rats fed with methylcholanthrene by gastric instillation has been made by Schultz, Jamison, Shay and Gruenstein (1954). They found an increase in the $\beta$-globulin protein component of rat serum and refer to the effect of lipid extraction which did not influence the $\beta$-globulin but produced instead a loss in the albumin area. Schultz *et al.* (1954) used boundary electrophoresis whilst the present results were obtained with zone electrophoresis, but it may be that as in the experiments described above the lipoprotein was spread over the globulin zone and hence lipid extraction would not produce an effect comparable with the effect on human serum, which has a sharp $\beta$-lipoprotein band.

Boundary electrophoretic examination has also been made by Cook, Griffin and Luck (1949) on the serum proteins of rats fed with carcinogenic azo dyes. Some of the azo dyes had little effect on the mobility of the serum proteins while *m'*-methyl-*p*-dimethylaminoazobenzene caused an increase in $\gamma$-globulin and a decrease in serum albumin. The authors state however that these changes may be non-specific since liver damage in humans can cause this type of protein change.

### SUMMARY.

There is evidence that benzpyrene added *in vitro* to human or animal serum is associated with the lipoprotein fraction. This evidence was obtained by (1) chromatographic examination on filter paper in which the fluorescent compound was associated with lipid material having a high $R_f$ value in water, and (2) zone electrophoresis in which the fluorescent compound migrated with the lipoprotein fraction of the serum.

There is also evidence that after intravenous injection of benzpyrene in rats and fowls the blood serum contains both unchanged and metabolised benzpyrene. Unchanged benzpyrene was associated with the globulin fraction of the serum and the metabolite with albumin.

The nature of the association of the hydrocarbon and its metabolite with protein has been discussed.

On zone electrophoresis human and fowl sera show the presence of a $\beta$-lipoprotein, but under the same conditions of electrophoresis, lipoprotein of rat and mouse sera appears to be unstable and forms a broad zone extending over the $\alpha$- to $\gamma$-globulin bands.

The author is indebted to Dr. P. R. Peacock for helpful discussion and for reading of the manuscript. He is also indebted to Mrs. R. H. Jack who carried out the lipid estimations and part of the electrophoresis analysis.

### REFERENCES.

BERENBLUM, I., CROWFOOT, D., HOLIDAY, E. R. AND SCHOENTAL, R.—(1943) *Cancer Res.*, **3**, 151.

*Idem* AND SCHOENTAL, R.—(1942) *Biochem. J.*, **36**, 92.—(1943) *Cancer Res.*, **3**, 145.—(1946) *Ibid.*, **6**, 699.

BLOOR, W. R.—(1943) 'Biochemistry of the Fatty Acids.' New York (Reinhold Publishing Corporation), p. 47.

CHALMERS, J. G.—(1953) *Biochem. J.*, **55**, 19.

*Idem* AND CROWFOOT, D.—(1941) *Ibid.*, **35**, 1270.

*Idem* AND KIRBY, A. H. M.—(1940) *Ibid.*, **34**, 1191.

*Idem* AND PEACOCK, P. R.—(1936) *Ibid.*, **30**, 1242.

COOK, H. A., GRIFFIN, A. C. AND LUCK, J. M.—(1949) *J. biol. Chem.*, **177**, 373.

COOK, J. W., LUDWICZAK, R. S. AND SCHOENTAL, R.—(1950) *J. chem. Soc.*, 1112.

DONIACH, I., MOTTRAM, J. C. AND WEIGERT, F.—(1943) *Brit. J. exp. Path.*, **24**, 1.

FLYNN, F. V. AND DE MAYO, P.—(1951) *Lancet*, ii, 235.

HEIDELBERGER, C. AND WEISS, S. M.—(1951) *Cancer Res.*, **11**, 885.

MILLER, E. C.—(1951) *Ibid.*, **11**, 100.

PEACOCK, P. R.—(1936) *Brit. J. exp. Path.*, **17**, 164.

SCHULTZ, J., JAMISON, W., SHAY, H. AND GRUENSTEIN, M.—(1954) *Arch. Biochem.*, **50**, 124.

WEIGERT, F., CALCUTT, G. AND POWELL, A. K.—(1947) *Brit. J. Cancer*, **1**, 405.

*Idem* AND MOTTRAM, J. C.—(1946*a*) *Cancer Res.*, **6**, 97.—(1946*b*) *Ibid.*, **6**, 109.

WIEST, W. G. AND HEIDELBERGER, C.—(1953*a*) *Ibid.*, **13**, 250.—(1953*b*) *Ibid.*, **13**, 255.

WOODHOUSE, D. L.—(1954) *Brit. J. Cancer*, **8**, 346.

WUNDERLY, CH. AND PEZOLD, F. A.—(1952) *Naturwissenschaften*, **39**, 493.